# Salviae miltiorrhizae and ligustrazine hydrochloride injection combined with mecobalamin for treating diabetic peripheral neuropathy: A protocol for systematic review and meta-analysis

**DOI:** 10.1097/MD.0000000000024103

**Published:** 2021-01-22

**Authors:** Zhiyuan Deng, Manjia Wang, Yaohua Fan, Min Liu

**Affiliations:** a Gaozhou Hospital of Traditional Chinese Medicine (Gaozhou Hospital of Traditional Chinese Medicine Affiliated to Guangzhou University of Chinese Medicine; b The First Clinical Medical College, Guangzhou University of Chinese Medicine, Guangzhou; c Shenzhen Hospital of Integrated Traditional Chinese and Western Medicine, Guangzhou University of Chinese Medicine; d Shenzhen Bao’an Traditional Chinese Medicine Hospital (Group), Guangzhou University of Chinese Medicine, Shenzhen; e The First Affiliated Hospital of Guangzhou University of Chinese Medicine, Guangzhou, China.

**Keywords:** diabetic peripheral neuropathy, mecobalamin, salviae miltiorrhizae and ligustrazine hydrochloride, systematic analysis

## Abstract

**Objective:**

Currently, it is unclear whether the salviae miltiorrhizae (Danshen Salvia) and ligustrazine hydrochloride (Chuanxiong Chuanxiong) (SMLH) injection combined with mecobalamin can improve diabetic peripheral neuropathy (DPN). We conducted a systematic analysis to evaluate the clinical effects of SMLH injection combined with mecobalamin for treating DPN.

**Methods:**

Seven databases, including PubMed, Embase, Cochrane Library, China National Knowledge Infrastructure (CNKI), Wan Fang Database (Wang Fang), Chinese Biomedical Literature Database (CBM), and VIP Database for Chinese Technical Periodicals (VIP) were searched for systematic literature retrieval. Each database was searched up to 2020 to identify randomized controlled trials on DPN treated with SMLH injection combined with mecobalamin. We used the RevMan 5.3 and Stata 14.0 software to assess the risk of bias in the included trials.

**Results:**

A total of 15 publications, including 1349 samples, were reviewed. The total effective rate of SMLH injection combined with mecobalamin was 31% higher than that of mecobalamin alone (95% confidence interval [CI] = 1.23–1.38; *P* < .00001). The experimental group showed a significant increase in the motor conduction velocity (MCV) of the peroneal nerve (weighted mean difference [WMD] = 4.81, 95% CI 3.53–6.09; *P* < .00001). In addition, SMLH injection combined with mecobalamin showed a statistical significant effect on the sensory conduction velocity (SCV) of the peroneal nerve (WMD = 5.03, 95% CI = 4.16–5.90; *P* < .00001), and MCV of the median nerve (WMD = 5.38, 95% CI = 4.05–6.72; *P* < .00001). The WMD for the change in SCV in the median nerve was 4.89 m/s (95% CI = 3.88–5.89; *P* < .00001). The *P*-values of the Egger and Begg tests were 0.967 and 0.961, respectively, indicating no publication bias. Subgroup and sensitivity analyses indicated that the results for MCV and SCV of the peroneal nerve and the median nerve were stable.

**Conclusion:**

SMLH injection combined with mecobalamin can improve DPN, compared with mecobalamin alone.

## 1 Introduction

Diabetes and its complications pose a serious threat to global health, which is expected to increase to 642 million^[1–2]^ by 2040; more than 90% of the affected patients have type 2 diabetes.^[3]^ An aging population and poor lifestyle habits, such as a high-fat/high-sugar diet and a sedentary lifestyle, aggravate the global spread of diabetes.^[4]^ Diabetic peripheral neuropathy (DPN) is a common complication of diabetes mellitus. In recent years, the prevalence of DPN has been increasing. According to current research, approximately 10% to 50% of diabetic patients will develop DPN,^[5]^ which is of serious concern.^[5]^ Pain, numbness, and an abnormal feeling in the limbs and trunk are common clinical manifestations of DPN. Severe cases include glove-like or sock-like sensory disorders and even muscle atrophy. Currently, approximately 50%–70% of non-traumatic amputations are performed due to DPN. Mortality of diabetic patients with neuropathy is expected to reach 25% to 50% in 5 to 10 years.^[6–7]^ The pathogenesis of DPN involves oxidative stress caused by long-term hyperglycemia, vascular injury,^[8–9]^ advanced glycation end products,^[10]^ and the polyol pathway.^[11]^ Early treatment is necessary and can delay the pathological process of DPN.^[6]^ Treatment of DPN includes general treatment, drug treatment, physical treatment, and surgical treatment. The drugs used for DPN include nimodipine; antioxidant α-lipoic acid; epalrestat, an aldose reductase inhibitor; and Chinese medicinal preparations. However, these drugs have limited effects when used alone. Therefore, it is necessary to study the effect of various therapeutic drug combinations.

Traditional Chinese medicine (TCM) is a traditional alternative therapy widely used in China. Based on the TCM theory, DPN can be classified as a “Bi syndrome,” which is mainly caused by blood stasis. Studies have established that Danshen reduces inflammatory reactions and vascular stress,^[12]^ while Chuanxiong promotes vasodilation and eliminates blood stasis.^[13–14]^ Salviae miltiorrhizae (Danshen Salvia) and ligustrazine hydrochloride (Chuanxiong Chuanxiong) (SMLH) injection, a combination of Danshen and Chuanxiong, can activate blood circulation, relieve pain, prevent platelet aggregation, dilate arterial vessels, reduce blood viscosity,and improve circulation.^[15–17]^ SMLH injection has a wide range of clinical applications and has shown beneficial effects on coronary heart disease, cerebral ischemia, cor pulmonale, and diabetic neuropathy,^[18–21]^ demonstrating that SMLH injection can increase blood circulation and alleviate symptoms. Therefore, SMLH injection may help improve DPN by promoting circulation and reducing inflammation. By far, most clinical studies have reported the effects of SMLH injection combined with mecobalamin. However, there is currently no systematic evaluation. In addition, the effects of SMLH injection may vary with the dosage and treatment course. This study aimed to evaluate the effects of SMLH injection combined with mecobalamin in the treatment of DPN, compared with mecobalamin alone.

## 2 Methods

### 2.1 Types of research

Published literature meeting all of the following criteria were included in this study:

(1) a randomized controlled trial (RCT);
(2) in terms of intervention measures, the experimental group was treated with SMLH injection combined with mecobalamin;
(3) mecobalamin was used in the control group, and other conditions were the same for both groups.

### 2.2 Research criteria

DPN was diagnosed based on the diabetes clinical practice guidelines.^[22]^ Exclusion criteria included acute diabetic complications, such as diabetic ketoacidosis and hyperosmolar coma. We excluded pregnant or lactating women, and patients with drug allergies or cancer. Furthermore, studies with incomplete or problematic data and repeated publications were excluded. Since this study used data that was previously published, ethical approval and patient consent were not required.

### 2.3 Types of intervention

The control group was treated with mecobalamin and other routine primary treatments, such as controlling blood glucose, lowering blood pressure, and regulating blood fat. The treatment group was treated with SMLH injection in addition to treatment administered to the control group.

### 2.4 Detection index

Detection indices included total effectiveness, motor conduction velocity (MCV), and sensory conduction velocity (SCV) of the peroneal nerve and the median nerve.

### 2.5 Search strategy

Databases including PubMed, Embase, Cochrane Library, China National Knowledge Infrastructure (CNKI), Wan Fang Database (Wan Fang), Chinese Biomedical Literature Database (CBM), and VIP Database for Chinese Technical Periodicals (VIP) were searched to retrieve data on RCTs related to interventions performed using SMLH injection combined with mecobalamin on DPN. The retrieval time limit was set to 2020. The search terms and keywords were: “salvia miltiorrhiza and ligustrazine,” “Danshen Chuanxiong,” “diabetic peripheral neuropathy,” and “DPN.” The method of combining free words and subject words was adopted in the retrieval, and the retrieval strategy was adjusted in accordance with the characteristics of different databases for multiple searches, to optimize the recall rate of documents.

### 2.6 Data processing and analysis

#### 2.6.1 Data screening

**Figure 1:**
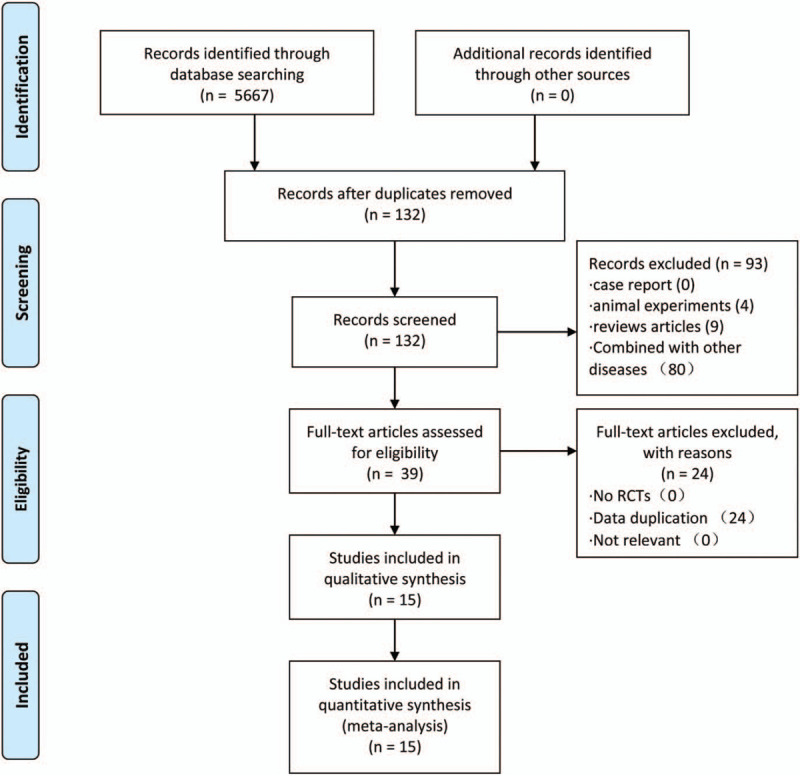
PRISMA flow chart.

Two reviewers independently screened the available publications by topic and abstract. Two reviewers extracted literature data, including basic information on patients, interventions, and experimental results. For points of inconsistency, a third reviewer was consulted. See Figure 1.

#### 2.6.2 Risk of bias analysis

The 2 reviewers independently screened the literature, extracted the basic data, assessed the bias risk, and compared them. In case of disagreement, the third reviewer was consulted. The extraction of basic data mainly included the following content:

(1) basic information included in the study, including the first author and publication time;
(2) baseline characteristics of the study object, including gender and age;
(3) intervention measures, including intervention mode, dosage, and treatment course;
(4) key elements of bias risk assessment; and
(5) outcome indicators and outcome measurement data.

Two evaluators evaluated the bias risk of RCTs according to the manual of the Cochrane system evaluators.

#### 2.6.3 Statistical methods

We used the statistical software RevMan 5.3 and Stata 14.0 from the Cochrane Collaboration. For dichotomous data, we used the risk ratios to analyze the statistics; for continuous data, we used the weighted mean difference (WMD)/standard mean difference (SMD) and the calculated 95% confidence intervals (CIs). The χ^2^ test was used to test the heterogeneity. If homogeneity was observed (*P* > .05; *I*^2^ < 50%) in the research results, the fixed effect model was used for meta-analysis; if there was a significant statistical heterogeneity (*P* ≤ .05; *I*^2^ ≥ 50%) between the research results, the random effect model was used for meta-analysis.

#### 2.6.4 Heterogeneity and subgroup analysis

If the heterogeneity between studies was large, we analyzed the source of heterogeneity using subgroup analysis. We conducted a subgroup analysis from the perspective of the dose, treatment course, and manufacturer.

#### 2.6.5 Publication bias

We used the Begg test and Egger test to detect publication bias. When *P* > .05, the deviation was considered not statistically significant.

## 3 Results

### 3.1 Research screening results

We searched 5667 articles and excluded 5535 articles unrelated to DPN. Ninety-three studies were excluded based on headlines and abstracts. A total of 84/93 studies were either based on combinations with other drugs and therapies or not compared with mecobalamin, and 9/93 studies were review articles. Thirty-nine articles met the storage requirements. Finally, only 15 studies met the inclusion criteria owing to repeated data in 24 cases.^[23–37]^

### 3.2 Research characteristics

The 15 selected studies included 1349 patients with DPN. All the publications were published in Chinese during 2010–2019. No multicenter experiments were reported (Table 1).

**Table 1:** Basic characteristics of included studies.

	T				C					
Included studies	Age (yr)	DM course (yr)	n	Intervention	Age (yr)	DM course (yr)	n	Intervention	Treatment Course	Outcomes
Zhu MM 2018	54.52 ± 10.31	8.66 ± 2.13	33	A	54.96 ± 10.45	8.21 ± 2.65	33	F	2weeks	(1) (2) (3) (4) (5)
Chang Y 2013	–	–	30	C	–	–	30	F	2weeks	(1) (2) (3) (4) (5)
Li H 2011	53.58 ± 6.72	–	38	B	55.14 ± 8.54	–	38	F	2weeks	(1) (2) (3) (4) (5)
Li JP 2011	35–78	2–38	60	C	34–75	2–35	26	F	2weeks	(1)
Dai LF 2010	54.5 ± 4.2	8.2 ± 3.4	41	A	55.3 ± 3.2	9.0 ± 2.5	39	F	3weeks	(1) (2) (3) (4) (5)
Liu L 2019	57.44 ± 12.30	9.65 ± 2.18	50	D	58.03 ± 12.78	9.88 ± 2.31	50	F	8weeks	(1) (2) (3) (4) (5)
Liang Y 2019	53 ± 5	3–15	35	C	54 ± 4	2–16	35	F	2weeks	(1) (2) (3) (4) (5)
Li TJ 2011	53–82	5–13	50	C	56–78	8–15	50	F	4weeks	(1) (2) (3) (4) (5)
Sun H 2013	57.6 ± 7.8	8.7 ± 2.3	57	C	59.1 ± 9.2	9.1 ± 2.1	57	F	3weeks	(1) (2) (3) (4) (5)
Zhang MX 2016	58.2 ± 6.8	4.5 ± 0.8	42	B	56.3 ± 5.5	5.2 ± 1.6	42	F	2weeks	(1) (2) (3) (4) (5)
Wang Y-H 2014	60.87 ± 8.84	16.9 ± 7.04	45	E	59.74 ± 9.34	15.49 ± 6.54	43	F	4weeks	(1)
Peng HX 2011	61.3 ± 8.6	8.3 ± 4.6	56	A	62.9 ± 8.9	8.5 ± 4.9	56	F	2weeks	(1) (2) (3)
Jiang ZM 2015	43–85	5–31	86	C	43–85	5–31	77	F	2weeks	(1) (2) (3) (4) (5)
Zhang YJ 2017	58.7 ± 7.9	8.4 ± 1.7	45	C	59.2 ± 8.5	9.1 ± 1.5	45	F	3weeks	(1) (2) (3) (4) (5)
Wang YH 2014	57.23 ± 6.61	13.53 ± 2.66	30	D	58.13 ± 7.05	12.83 ± 2.53	30	F	8weeks	(1) (2) (3) (4) (5)

Two-thirds of the trials (10 trials) were small sample studies (sample size < 100). The average treatment course in these studies ranged from 2–8 weeks. The treatment course was 2 weeks in 8 trials, 3 weeks in 3 trials, 4 weeks in two trials, and 8 weeks in two trials. All studies evaluated the total effective rate, 13 evaluated the MCV and SCV of the peroneal nerve, and 12 evaluated the MCV and SCV of the median nerve.

### 3.3 Bias risk

**Figure 2:**
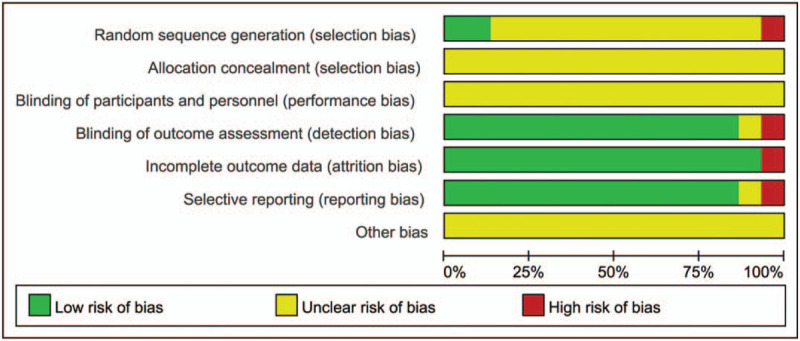
Risk of bias graph.

**Figure 3:**
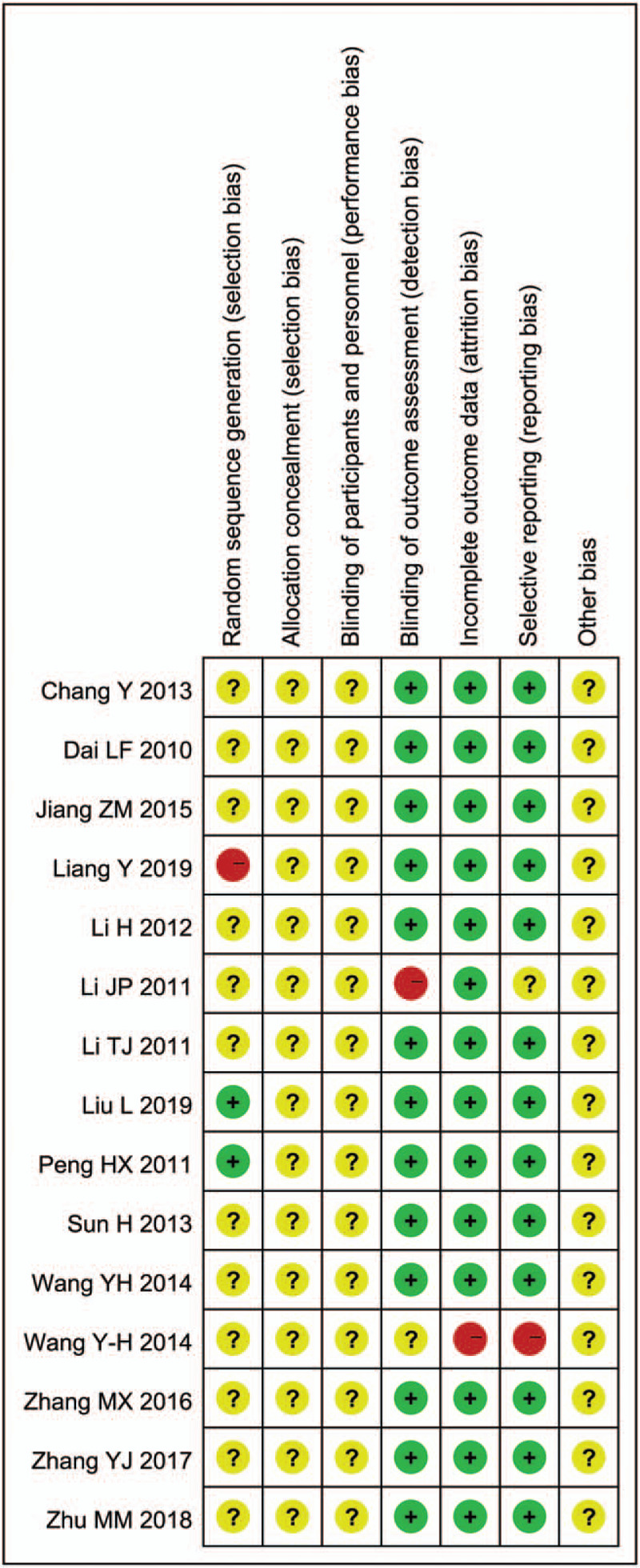
Risk of bias summary.

Based on the methodological quality chart (Figs. 2 and 3), only 2 papers mentioned the specific randomization method, 1 article was grouped according to the order of admission with a high risk, and the rest lacked clarity. Adverse reactions were reported in 2 articles. Blinding details were not mentioned in any trial.

### 3.4 Statistical analysis results

#### 3.4.1 Total effective rate

**Figure 4:**
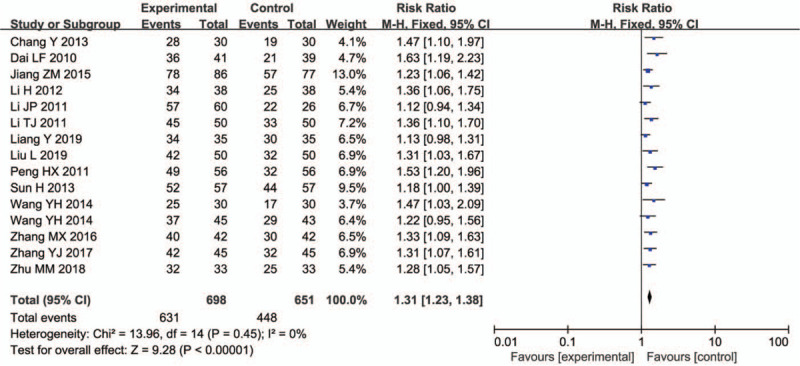
Total effective rate.

Fifteen trials, including 1349 samples, reported the total effective rate. There was no statistical significance in the heterogeneity test; hence, a fixed effect model was used. The total effective rate was 31% higher than that of the control (95% CI = 1.23–1.38; *P* < .00001) (Fig. 4).

#### 3.4.2 Peroneal nerve MCV

**Figure 5:**
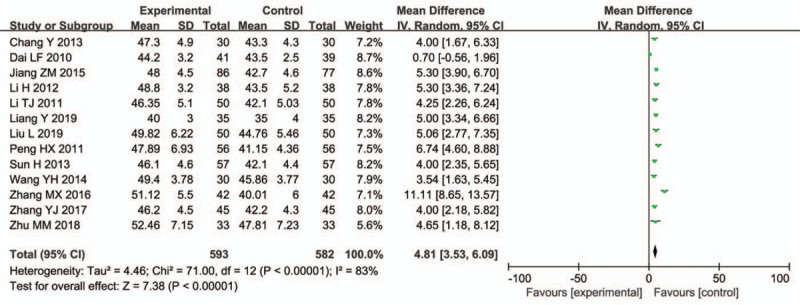
Peroneal nerve MCV.

Thirteen trials (1175 participants) evaluated the peroneal nerve MCV. Since the heterogeneity was high, we used the random effect model for analysis. The peroneal nerve MCV treated with combined medication showed an increase (WMD = 4.81, 95% CI = 3.53–6.09; *P* < .00001) (Fig. 5).

#### 3.4.3 Peroneal nerve SCV

**Figure 6:**
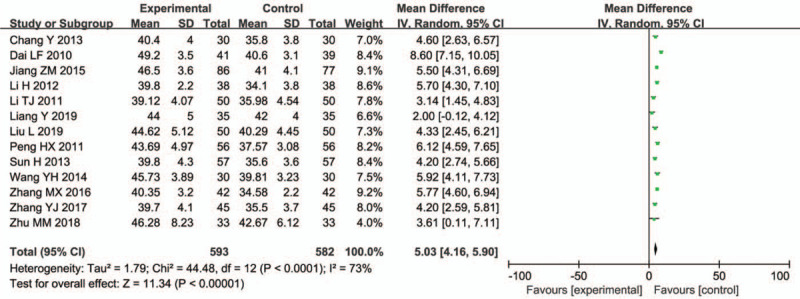
Peroneal nerve SCV.

Thirteen trials (1175 participants) evaluated the peroneal nerve SCV and found a significant difference (WMD = 5.03, 95% CI = 4.16–5.90; *P* < .00001) (Fig. 6).

#### 3.4.4 Median nerve MCV

**Figure 7:**
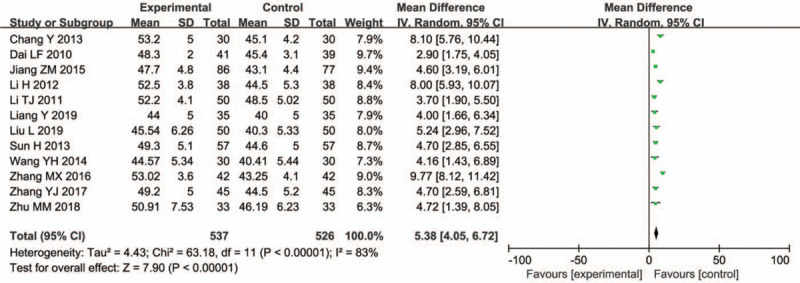
Median nerve MCV.

Twelve trials (1063 participants) reported on the median nerve MCV. The combined medication group showed a significant increase (WMD = 5.38, 95% CI = 4.05–6.72; *P* < .00001) (Fig. 7).

#### 3.4.5 Median nerve SCV

**Figure 8:**
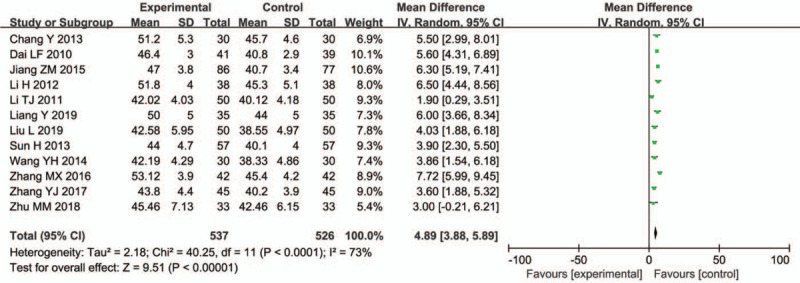
Median nerve SCV.

Twelve studies (1063 participants) reported on the median nerve SCV. In a joint analysis, the WMD in the median nerve SCV change was 4.89 m/s (95% CI = 3.88–5.89; *P* < .00001; *I*^2^ = 73%) (Fig. 8).

#### 3.4.6 Subgroup analysis of MCV and SCV of the peroneal nerve and the median nerve

The heterogeneity decreased on subgroup analysis. Subgroup analysis showed that for MCV of the peroneal nerve, the estimated effect of the 15 mL dose was greater than that of other doses. However, this effect did not increase with treatment. In addition, the estimated effect of the Guizhou Baite group was greater than that of the other groups. For SCV of the peroneal nerve, the estimated effect was highest for the 20 mL dose. The group with a 3-week treatment course had the highest expected effect. For MCV and SCV of the median nerve, the group with a 2-week treatment course and the 15 mL dose had the highest expected effect. For most results, the drug produced by Guizhou Baite had a better expected effect than the others (Tables 2 and 3).

**Table 2:** Subgroup analyses of the effect of salviae miltiorrhizae and ligustrazine hydrochloride injection for improving peroneal nerve MCV and SCV.

	Peroneal nerve MCV			Peroneal nerve SCV		
Subgroup	Studies	Patient	MD (95% CI)	Studies	Patient	MD (95CI)
Dose						
Intravenous infusion 20 mL	3^[23,27,34]^	258	3.94 (-0.38–8.25)	3^[23,27,34]^	258	6.49 (4.05–8.94)
Intravenous infusion 15mL	2^[25,32]^	160	8.15 (2.46–13.85)	2^[25,32]^	160	5.74 (4.84–6.64)
Intravenous infusion 10mL	6^[24,29–31,35–36]^	597	4.55 (3.84–5.26)	6^[24,29–31,35–36]^	597	4.09 (3.13–5.05)
Acupoint injection 1 mL	2^[28,37]^	160	4.16 (2.69–5.63)	2^[28,37]^	160	5.15 (3.59–6.70)
Test for subgroup differences: p = 0.60, I^2^ = 0%				Test for subgroup differences: *P* = .06, *I*^2^ = 60%		
Treatment duration						
2 weeks	7^[23–25,29,32,34,35]^	631	5.99 (4.46–7.51)	7^[23–25,29,32–35]^	631	5.08 (4.18–5.99)
3 weeks	3^[27,31,36]^	284	2.83 (0.48–5.19)	3^[27,31,36]^	284	5.68 (2.75–8.61)
4 weeks	1^[30]^	100	4.25 (2.26–6.24)	1^[30]^	100	3.14 (1.45–4.83)
8 weeks	2^[28,37]^	160	4.16 (2.69–5.63)	2^[28,37]^	160	5.15 (3.59–6.70)
Test for subgroup differences: p = 0.12, I^2^ = 48%				Test for subgroup differences: *P* = .19, *I*^2^ = 37.8%		
Manufactor						
Guizhou Baite	7^[23,25,30–32,34–35]^	715	5.84 (4.25–7.44)	7^[23,25,29–32,34–35]^	715	5.09 (4.30–5.87)
Jilin Sichang	2^[28,36]^	190	4.41 (2.98–5.83)	2^[28,36]^	190	4.26 (3.03–5.48)
Other	4^[24,27,29,37]^	270	3.24 (1.05–5.43)	4^[24,27,29,37]^	270	5.35 (2.58–8.12)
Test for subgroup differences: p = 0.15, I^2^ = 48.1%				Test for subgroup differences: *P* = 0.50, *I*^2^ = 0%		

**Table 3:** Subgroup analyses of the effect of salviae miltiorrhizae and ligustrazine hydrochloride injection for improving peroneal nerve MCV and SCV.

Subgroup	Median nerve MCV			Median nerve SCV		
	Studies	Patient	MD (95% CI)	Studies	Patient	MD (95CI)
Dose						
Intravenous infusion 20 ml	2^[23,27]^	146	3.11 (1.97–4.25)	2^[23,27]^	146	4.73 (2.33–7.14)
Intravenous infusion 15ml	2^[25,32]^	160	9.00 (7.28–10.72)	2^[25,32]^	160	7.21 (5.89–8.54)
Intravenous infusion 10ml	6^[24,29–31,35–36]^	597	4.86 (3.77–5.96)	6^[24,29–31,35–36]^	597	4.49 (2.97–6.01)
Acupoint injection 1ml	2^[28,37]^	160	4.80 (3.05–6.54)	2^[28,37]^	160	3.95 (2.38 –5.53)
Test for subgroup differences: p < 0.001, I^2^ = 90.4%				Test for subgroup differences: *P* = 0.007, *I*^2^ = 75.2%		
Treatment duration						
2 weeks	6^[23–25,29,32,34,35]^	519	6.61 (4.52–8.70)	6^[23–25,29,32,34,35]^	519	6.23 (5.27–7.18)
3 weeks	3^[27,31,36]^	284	3.88 (2.56–5.21)	3^[27,31,36]^	284	4.46 (3.16–5.76)
4 weeks	1^[30]^	100	3.70 (1.90–5.50)	1^[30]^	100	1.90 (0.29–3.51)
8 weeks	2^[28,37]^	160	4.80 (3.05–6.54)	2^[28,37]^	160	3.95 (2.38–5.53)
Test for subgroup differences: p = 0.13, I^2^ = 46.6%				Test for subgroup differences: *P* < 0.001, *I*^2^ = 86.6%		
Manufactor						
Guizhou Baite	6^[23,25,30–32,35]^	603	5.95 (3.88–8.03)	6^[23,25,30–32,35]^	603	4.97 (3.14–6.80)
Jilin Sichang	2^[28,36]^	190	4.95 (3.40–6.50)	2^[28,36]^	190	3.77 (2.43–5.11)
Other	4^[24,27,29,37]^	270	4.70 (2.38–7.02)	4^[24,27,29,37]^	270	5.36 (4.42–6.31)
Test for subgroup differences: p = 0.68, I^2^ = 0%				Test for subgroup differences: *P* = 0.16, *I*^2^ = 45.3%		

### 3.5 Publication bias

The P-values of the Egger and Begg tests were 0.967 and 0.961, respectively, indicating no publication bias.

### 3.6 Sensitivity analysis

Sensitivity analysis by sample size and risk of bias did not show any significant effect on the main outcomes of the nerve conduction velocity (Table 4).

**Table 4:** Sensitivity analyses by sample size and risk of bias.

	Study removed from the primary meta-analysis	Number of included studies	RR/WMD (95% CI)	Heterogeneity
Sample size				
Total effective rate	10^[23–27,29,32–33,36–37]^	5^[28,30,31,34,35]^	1.30 (1.19–1.42)	*P* = 0.43; *I*^2^ = 0%
Peroneal nerve MCV	8^[23–25,27,29,32,36–37]^	5^[26,30,31,34,35]^	5.00 (4.12–5.88)	*P* = 0.32; *I*^2^ = 14%
Peroneal nerve SCV	8^[23–25,27,29,32,36–37]^	5^[28,30,31,34,35]^	4.73 (3.72–5.74)	*P* = 0.07; *I*^*2*^ = 54%
Median nerve MCV	8^[23–25,27,29,32,36–37]^	4^[28,30,31,35]^	4.50 (3.62–5.38)	*P* = 0.75; *I*^*2*^ = 0%
Median nerve SCV	8^[23–25,27,29,32,36–37]^	4^[28,30,31,35]^	4.08 (2.02–6.14)	*P* = 0.0001; *I*^2^ = 86%
Risk of bias				
Total effective rate	3^[26,29,33]^	12^[23–25,27,28,30–32,34–37]^	1.34 (1.26–1.43)	*P* = 0.77; *I*^2^ = 0%
Peroneal nerve MCV	1^[29]^	12^[23–25,27,28,30–32,34–37]^	4.80 (3.40–6.20)	*P* < 0.0001; *I*^2^ = 84%
Peroneal nerve SCV	1^[29]^	12^[23–25,27,28,30–32,34–37]^	5.26 (4.44–6.09)	*P* = 0.0002; *I*^2^ = 69%
Median nerve MCV	1^[29]^	11^[23–25,27,28,30–32,35–37]^	5.50 (4.07–6.93)	*P* < 0.0001; *I*^2^ = 84%
Median nerve SCV	1^[29]^	11^[23–25,27,28,30–32,35–37]^	4.79 (3.72–5.87)	*P* < 0.0001; *I*^2^ = 75%

## 4 Discussion

The study included 15 RCTs involving 1349 patients with DPN. The analysis revealed that SMLH injection combined with mecobalamin led to an increase in the total effective rate (by 31%), MCV, and SCV of the peroneal nerve and the median nerve. In the subgroup analysis, there was no significant difference in the peroneal nerve MCV in the subgroup with a dose of 20 mL. This may be due to the lack of sample size and the interference of small sample research. However, other doses and treatment courses were stable. The effect value of 15 mL was the highest; however, the sample size was small. The effect value of 10 mL was the second highest, and with a certain sample size, the results were reliable. The results supported that SMLH injection combined with mecobalamin may improve DPN.

An important advantage of this study was a comprehensive literature search, including some recently published experiments. These studies improved the accuracy of the results. In addition, the sensitivity analysis of the peroneal nerve MCV, peroneal nerve SCV, median nerve MCV, and median nerve SCV, which are important evaluation indices of DPN, showed that the preliminary results were stable.

Both salviae miltiorrhiza radix (Danshen) and chuanxiong rhizoma (Chuanxiong) are used in the treatment of ischemic vascular diseases owing to their vascular protective effects.^[13–14,38]^ Danshen is used for its anti-oxidative anti-atherosclerotic, and anti-inflammatory effects.^[12]^ In addition, Danshen exhibits anti-diabetic effects by treating macrovascular and microvascular diseases in preclinical experiments and clinical trials, through an improvement in redox homeostasis and inhibition of apoptosis and inflammation via the regulation of Wnt/β-catenin,^[39]^ TSP-1/TGF-β1/STAT3,^[40–42]^ and JNK/PI3K/Akt^[43]^. Similarly, Chuanxiong promotes blood vessel dilation and eliminates blood stasis. According to previous research,^[44]^ Danshen has an obvious anti-inflammatory effect on bone marrow macrophages. However, it has minimal effect on human umbilical vein endothelial cells. On the contrary, Chuanxiong has a considerable anti-inflammatory effect on human umbilical vein endothelial cells, but no anti-inflammatory effect on bone marrow macrophages. In toll-like receptor-activated inflammatory responses, SMLH shows a “multi-component multi-target” effect, in the presence of a complementary mechanism. Therefore, SMLH injection combined with mecobalamin can reduce inflammatory reactions through multiple pathways and mechanisms, leading to an improvement in DPN.

There were some limitations to this study. First, most of the included studies had a small sample size, with minimal reports on the implicit and specific random methods of the distribution scheme. A selective bias could be present; the methods were not blinded, which could lead to a measurement bias. Second, all the included publications were in Chinese, which may lead to the risk of bias and increase the limitations of conclusions. This is because Chinese literature tends to publish positive results, while negative results are difficult to publish or do not receive sufficient attention. This could lead to a publication bias, resulting in overestimation or underestimation of the results. Furthermore, most of the studies were completed in 2 weeks, and few studies were conducted for longer than 2 weeks. DPN is a long-term chronic disease that requires long-term treatment. Studies that investigated the treatment course for longer than 2 weeks may require further verification of the effectiveness of SMLH injection combined with mecobalamin.

## 5 Conclusion

SMLH injection combined with mecobalamin has a better curative effect and better tolerance for patients with DPN, compared with mecobalamin alone. However, the small samples included in some studies may reduce the strength of the evidence. In addition, larger samples, better study design, and RCTs are needed to determine whether SMLH injection combined with mecobalamin, in a prolonged treatment cycle and at other doses, has a perpetual role in improving DPN.

## Author contributions

Zhiyuan Deng and Min Liu chaired the design of the study. Manjia Wang and Yaohua Fan were responsible for drafting the paper. Manjia Wang and Yaohua Fan proposed retrieval strategies. Zhiyuan Deng and Manjia Wang were responsible for data collection. All authors approved the final manuscript.

**Conceptualization:** Zhiyuan Deng.

**Data integration:** Zhiyuan Deng, Manjia Wang.

**Formal analysis:** Manjia Wang

**Methodology:** Yaohua Fan.

**Resources:** Zhiyuan Deng, Manjia Wang.

**Review and editing:** Min Liu

**Software operation:** Zhiyuan Deng.

**Original manuscript writing:** Zhiyuan Deng, Min Liu.
